# Blood Conservation and Hemostasis Management in Pediatric Cardiac Surgery

**DOI:** 10.3389/fcvm.2021.689623

**Published:** 2021-08-19

**Authors:** Roby Sebastian, M. Iqbal Ahmed

**Affiliations:** Department of Anesthesiology and Pain Management, UT Southwestern Medical Center, Children's Medical Center, Dallas, TX, United States

**Keywords:** blood conservation, transfusion risks, pediatric cardiac surgery, hemostasis, blood product, blood transfusion

## Abstract

Pediatric cardiac surgery is associated with significant perioperative blood loss needing blood product transfusion. Transfusion carries serious risks and implications on clinical outcomes in this vulnerable population. The need for transfusion is higher in children and is attributed to several factors including immaturity of the hemostatic system, hemodilution from the CPB circuit, excessive activation of the hemostatic system, and preoperative anticoagulant drugs. Other patient characteristics such as smaller relative size of the patient, higher metabolic and oxygen requirements make successful blood transfusion management extremely challenging in this population and require meticulous planning and multidisciplinary teamwork. In this narrative review we aim to summarize risks and complications associated with blood transfusion in pediatric cardiac surgery and also to summarize perioperative coagulation management and blood conservation strategies.

## Introduction

Pediatric cardiac surgery on cardiopulmonary bypass (CPB) is associated with significant bleeding and blood transfusion requirements. Bleeding after pediatric cardiac surgery is generally related to a combination of several factors and include immaturity of the hemostatic system, hemodilution from the CPB circuit, excessive activation of the hemostatic system, and potentially preoperative anticoagulant drugs. The number of children undergoing cardiac surgery has significantly increased in the past few decades and blood transfusion management has become an integral aspect of intra-operative patient care and is vital to successful outcomes. However, there is an increasing concern regarding the risks and complications associated with homologous blood transfusion in the pediatric cardiac surgical population. Hence there is an effort to develop various perioperative strategies both to avoid and restrict blood transfusions in this population.

This narrative review is based on a targeted search of the literature databases including PubMed, Embase, Medline and the Cochrane Database of Systematic Reviews. Search terms combining *transfusion risk, pediatric cardiac surgery, blood conservation, postoperative bleeding, blood products, cardiopulmonary bypass* were used. References cited in the retrieved literature were also examined for relevance.

## Epidemiology and Cost

In the United states the cost of health care is anticipated to grow at a rapid pace causing tremendous burden on the healthcare system, patients and the government. The Center for Medicare and Medicaid Services estimates that the national health spending is projected to grow at an average rate of 5.5 percent per year for 2018–27 and to reach $6.0 trillion by 2027. Health spending is projected to grow 0.8 percentage point faster than Gross Domestic Product (GDP) per year over the 2018–27 period; as a result, the health share of GDP is expected to rise from 17.9 percent in 2017 to 19.4 percent by 2027.

According to the National Blood Collection and Utilization Survey Report, 14.9 million RBC units were transfused in the United States in 2009 (1). A 2007 study estimated that the activity-based cost of transfusion for a single RBC unit in a surgical patient was calculated to be between $726 and $1,183 (data from the two US facilities) (2). If the mean value for the activity-based cost of an RBC transfusion from the US facilities is used for calculation ($954.50), then the annual cost of RBC transfusions in the United States exceeds $14.2 billion. The underlying assumption of this calculation is, however, that all transfused populations are comparable to the surgical patient populations used in the activity-based cost analysis. This assumption, of course, is not true. The costs associated with the care of patients who have the wide array of disease processes that require transfusion support are much more variable (3).

It is estimated that between 10 and 20% of the total erythrocytes transfused in the United States annually are given to cardiac surgical patients and within this group the need for transfusion in pediatric cardiac surgery is even higher (4, 5). Approximately 50% of the more than 7 million units of platelets transfused annually are to patients undergoing cardiac surgery and use of platelet transfusion continues to grow.

World health organization's recognition of blood and blood products as essential medicine emphasizes their crucial importance in any national healthcare system (6). Blood transfusion continues to be an important resource of every health care system and globally there is a significant gap between need and supply particularly in the low and middle-income countries (7). While government and healthcare systems implement policies to meet these demands, it's imperative that as healthcare providers we continue to apply evidence-based blood conservation strategies at every level to reduce this burden.

## Transfusion and Clinical Outcomes

Many factors determine the need for blood transfusion in pediatric cardiac surgery. Hemoglobin levels, lactates, arteriovenous oxygen differences and clinical picture are some of the factors that drive the decision to transfuse. Apart from direct risks associated with transfusion, it has a significant impact on clinical outcomes.

A retrospective single-center cohort study by Kneyber et al. showed that RBC transfusion was independently associated with increased mortality, prolonged duration of mechanical ventilation, increased need for infusion of vasoactive agents, and increased duration of pediatric intensive care unit stay. It also showed higher mortality rates among patients with multiple transfusions (8). Lacroix et al. in a landmark study comparing restrictive (hemoglobin threshold of 7 g per deciliter) vs. liberal (threshold of 9.5 g per deciliter) transfusion strategy showed a 96% reduction in the number of patients who had any transfusion exposure and a 44% decrease in the number of red-cell transfusions administered, without significant changes in mortality or morbidity between the two groups (9). Another prospective randomized study suggests that infants and neonates who require cardiac surgical repair or palliation can be safely managed during the immediate postoperative period using a conservative RBC transfusion strategy without significantly affecting oxygen delivery, mean/peak arterial lactate, lactate clearance, or estimated arteriovenous oxygen difference or clinical outcomes. This suggests that a conservative RBC transfusion protocol immediately after operation is possible, even in these vulnerable patients (10).

A study looking into perioperative risk factors for surgical site infections (SSI) in pediatric cardiac surgery showed that receipt of three or more units of red blood cells was independently associated with subsequent development of organ space SSI (11). This is in similarity to other studies in adult cardiac surgery showing an increased association between SSI and transfusion.

## Risks of Transfusion

Risks associated with transfusion can be classified into infective and non-infective causes. The non-infective causes can be further divided into immune and non-immune mediated complications (**Table 2**). The non-infective causes account for majority (>80%) of the complications commonly encountered in pediatric cardiac surgery.

### Infectious Diseases

Historically the infective causes have been the greatest cause for concern among patients. There was considerable alarm and anxiety in the 1980's with the recognition of transfusion-associated acquired immunodeficiency syndrome (AIDS) and transmission of hepatitis C virus (HCV). However, with better screening and testing methods there has been remarkable successes in reducing transmission of known viral agents and rapid responses to emerging infectious diseases that are documented to be transmitted by blood transfusion. Prior to current viral testing procedures, viruses presented the greatest risk for transfusion-transmitted infection (Table 1). However, at the present time, bacterial contamination of blood products presents the greatest risk and is reported to be the second most frequently reported cause of blood transfusion-related death after hemolytic reactions, and it accounts for more than 10% of transfusion-associated deaths in the USA (12–18). The greatest risk for bacterial infection is consistently demonstrated for platelet concentrates stored at room temperature with an incidence of 1:2000–3000 platelet transfusions. Transmission of other infections such as malaria and protozoal organisms tend to be confined to certain geographical regions. However, with ever increasing international travel there is a potential to acquire these diseases through transfusion outside their geographical boundaries. Clinicians can also play a great role in the reduction of secondary contamination of blood products by following current requirements that all blood products be transfused within 4 h of the blood product unit being started or returned to the blood bank within 30 min if it is not to be used immediately.

**Table 1:** Common infections associated with transfusion.

**Organisms**
**Viruses** • Hepatitis A, B, C, D • HIV 1 & 2 • HTLV 1 & 2 • CMV • Others: Parvovirus, West-Nile
**Bacteria** • Gram positive • *Staphylococcus* • *Streptococcus* • *Bacillus* • Gram negative • *Escherichia coli* • *Acinetobacter*, • *Klebsiella*
**Parasites** • Malaria • Babesiosis • Toxoplasmosis • Chagas
**Prions** • Variant Creutzfeldt-Jakob

*HTLV, Human T-cell lymphotropic virus; HIV, Human immunodeficiency Virus; CMV, Cytomegalovirus*.

### Non-infectious Causes

The non-infectious causes account for most of the transfusion associated mortality and serious morbidity. Transfusion related acute lung injury (TRALI), transfusion associated circulatory overload (TACO) and hemolytic transfusion reactions (HTRs) account for majority of them. Although rare, transfusion related immunomodulation (TRIM) and transfusion associated graft vs. host disease (TAGVHD) are serious risks (Table 2).

**Table 2:** Non-infectious complications of transfusion.

**Immune mediated**	**Non-immune mediated**
• Transfusion-related acute lung injury (TRALI) • Hemolytic transfusion reaction • Febrile non-hemolytic transfusion reaction • Allergic transfusion reaction • Alloimmunization • Transfusion-associated graft vs. host disease (TAGVHD) • Transfusion-related immunomodulation (TRIM)	• Transfusion-associated circulatory overload (TACO) • Metabolic abnormalities • Coagulation derangements from massive transfusion • Iron overload

#### Transfusion Related Acute Lung Injury

Transfusion related acute lung injury is defined as new acute lung injury (ALI) occurring during or within 6 h after a transfusion (17). The pathophysiology appears to be predominantly an antibody-mediated process resulting from the presence of antineutrophil and/or anti-HLA antibodies present in the donor plasma (19, 20). The diagnosis of TRALI is solely based on its clinical presentation and depends on a high level of suspicion and vigilance at the bedside given that it is a commonly underreported entity (21). Respiratory insufficiency, hypoxemia and bilateral fluffy infiltrates consistent with pulmonary edema on imaging are the hallmarks of clinical presentation. The true incidence of TRALI is variable and is thought to be often underreported especially in cardiac surgical population due to other confounding factors such as the effect of CPB on the lungs, nature of the surgical repair, postoperative atelectasis etc.

#### Transfusion Associated Circulatory Overload

TACO is an underreported complication of transfusion and is often avoidable. It is caused by an excessive quantity of transfused blood components or an increased rate of transfusion. The National Healthcare Safety Network definition requires- new onset, or acute exacerbation of three or more of the following, within 6 h of transfusion: respiratory distress, raised brain natriuretic peptide (BNP or NT-pro-BNP), increased central venous pressure, left heart failure, positive fluid balance, or pulmonary edema (22). Although TACO is less common in children, the pediatric cardiac surgical patients are often more vulnerable due immaturity of the cardiopulmonary and circulatory system.

#### Hemolytic Transfusion Reactions

HTRs occur when there is immunologic incompatibility between a transfusion recipient and the red blood cells (RBCs) from the blood donor. Hemolysis is the rupture of red blood cells and can occur intravascularly (circulation) or extravascularly (reticuloendothelial system). Based on the timing of the reaction they are classified as acute HTR that happen within 24 h of transfusion and delayed HTRs that happen after 24 h. Delayed HTRs usually manifest 1–2 weeks after transfusion, often tends to mild and goes undiagnosed. ABO incompatibility is the most common cause of acute HTRs and is commonly the result of clerical or procedural error. Clinically the classic triad consisting of fever, flank pain, and red or brown urine is not often seen. HTRs can range in severity from mild, clinically inapparent hemolysis weeks after the transfusion to rapid instantaneous, massive, intravascular hemolysis that may cause disseminated intravascular coagulation, shock, renal failure and even death. The prevalence of acute HTRs has been estimated at ~1 in 70,000 per blood product transfused (23).

## Blood Conservation Strategies

The pediatric surgical population is a challenging group due to many of its unique characteristics such as smaller relative size of the patient with respect to the CPB circuit, immature blood vessels and coagulation systems, higher metabolic needs which means higher oxygen requirements and longer duration on CPB and surgery. These characteristics not only increase the incidence of the resulting complications, but also their severity when they occur. These unique characteristics also make successful blood transfusion management extremely challenging in this population. Hence blood conservation techniques require meticulous planning and multidisciplinary teamwork throughout the various stages of the perioperative continuum. Many institutions over have developed protocols and guidelines for efficient conduct of these strategies and is summarized in Table 3.

**Table 3:** Perioperative blood conservation strategies.

**Preoperative** • Preoperative autologous donation • Erythropoietin supplementation • Iron supplementation • Minimal blood draw for investigations (both quantity and repetitive lab draws)
**Intraoperative** • Multidisciplinary planning • Strict fluid management to minimize hemodilution • Acute normovolemic hemodilution • Perfusion strategies - Optimal circuit for CPB - Retrograde autologous prime - Ultrafiltration (continuous zero-balance and modified ultrafiltration) - Cell salvage • Surgical expertise • Pharmacological agents - Antifibrinolytics - Tranexamic acid - Recombinant factor VII A - FEIBA • Effective hemodynamic management
**Postoperative** • Pharmacological agents • Effective hemodynamic management

### Jehovah's Witness

The JW population present significant challenges in transfusion management in pediatric cardiac surgery. Patients of JW faith refuse allogeneic blood products, viewing blood transfusion a direct violation of god's will and their faith. JW refuse the four main components of blood including red blood cells, white blood cells, plasma, and platelets and also autologous blood that has been removed from the body. The acceptance of other products such as factor concentrates, albumin or erythropoietin is subjective and needs to be discussed during preoperative planning (24). Some JWs consent to receive autologous blood if it remains in continuity with the body and the intraoperative setup should comply to satisfy these needs. Thorough multidisciplinary planning, preoperative optimization of anemia, intraoperative surgical technique, perfusion strategies and post-operative ICU management all contribute to satisfactory outcomes. The research and benefits of strategies used in these JW patients have become the standard of practice even in the non-JW population in many institutions.

### High Risk Patients

It is key to identify patients who are at a higher risk of bleeding and plan accordingly. Patients with cyanotic CHD, coagulations disorders, those on anticoagulation medications and neonates have a higher bleeding risk.

Secondary polycythemia is common in patients with cyanotic CHD, this can cause thrombocytopenia, platelet function abnormalities, disseminated intravascular coagulation, decreased production of coagulation factors, impaired liver function, vitamin K deficiency and primary fibrinolysis (25).

Opinion and recommendation of a hematologist should be sought for coagulation management of patient with primary coagulation disorders.

Neonates by the virtue of their immature coagulation system need a planned and strategic approach to postoperative hemostasis.

Children on antithrombotic medications undergoing cardiac surgery have a higher risk of perioperative bleeding due to the nature of the surgery, duration and the indication for anticoagulation (26). With improving pediatric cardiac surgery outcomes, there is an increased number of children presenting with antithrombotic medications due an increased risk of venous thromboembolism (VTE). These patients are almost always fall into the highest risk category. See Table 4 for commonly used antithrombotic medications. The use of newer oral antithrombotic are rare in children and can occasionally be seen in older and adult congenital cardiac patients.

**Table 4:** Antithrombotic medications.

**Antiplatelet drugs** - Aspirin (most common) - Clopidogrel
**Heparin** - Unfractionated heparin - Low molecular weight heparin (LMWH) ∘ Enoxaparin ∘ Dalteparin ∘ Tinzaparin
**Vitamin K antagonist (VKA)** - Warfarin (most common) - Acenocoumarol
**Newer oral antithrombotic** - Xa inhibitors ∘ Rivaroxaban ∘ Apixaban ∘ Edoxaban - Thrombin inhibitor ∘ Dabigatran

Planning cessation of antithrombotic treatment for elective cardiac surgery and reversal for emergency surgery should be done meticulously and guidelines for this is summarized in Table 5. The risk of thrombosis should be weighed against that of excessive bleeding and should be discussed in detail with hematology, cardiology and cardiac surgery teams.

**Table 5:** Preoperative management of antithrombotic medications.

**Medication**	**Elective surgery**	**Emergency surgery**
Aspirin	Stop for 5 days	Platelet transfusion
Warfarin	Stop 4 days Bridge to LMWH from day 3 to 1 Check INR	PCC, FFP
LMWH	Stop 24-h prior	Protamine (partial reversal only)
Xa inhibitor	Stop for 48 h	Andexanet alpha, PCC, FFP
Thrombin inhibitor	Stop for 48 h	Darucizumab, PCC, FFP

*PCC, prothrombin complex concentrate; FFP, fresh frozen plasma; LMWH, low molecular weight heparin; INR, international normalized ratio*.

#### Preoperative Strategies

##### Blood Donation

Preoperative autologous donation (PAD) has been studied extensively in the pediatric cardiac surgical population. Matsuda et al. looked at PAD in children <20 kgs between the ages 3 and 9 years undergoing open heart surgery. PAD was done up to 6+/-2 times preoperatively removing 5–10 ml/kg on each occasion. Results showed that the study group did not receive any allogeneic blood, compared with 80% of controls (27). Despite other studies also showing favorable results with PAD the process of blood collection including multiple vascular injections, difficult access, complications associated with the procedure itself has made PAD less favorable in children. Moreover, better intraoperative blood conservation strategies on CPB and studies questioning the true benefits of PAD has made it a less desirable strategy (28, 29).

##### Erythropoietin (Ep) and Iron Supplements

Preoperative erythropoietin and iron supplements have been extensively studied and shown to be effective in increasing hemoglobin levels and to decrease the use of blood and blood products preoperatively. A large meta-analysis and systematic review involving 32 trials (9 cardiac surgery studies) looking at preoperative erythropoietin and iron supplementation showed a significant reduction in homologous blood transfusion without risk of thromboembolic complications (30). Majority of these studies involved varying and multiple doses of Ep and Iron which may not be feasible in pediatric cardiac surgery. However, even a single dose might be effective in increasing hemoglobin and potentially decreasing transfusion requirements (31).

#### Intraoperative Strategies

##### Acute Normovolemic Hemodilution

ANH is a process wherein blood is removed from the patient, anticoagulated with a citrate solution, and stored. This blood is then transfused back to the patient following separation from CPB and heparin reversal with protamine. The primary objective of ANH is to protect platelets and coagulation factors from the untoward effects of cardiopulmonary bypass and offers an important autologous blood product that improves hemostasis at the conclusion of surgery. Traditionally during ANH, blood is replaced with crystalloid or colloid in a 3:1 ratio. This may not be applicable in children as it may result in results in both excessive hemodilution and unacceptably low hematocrit on CPB. ANH may have to be modified by avoiding fluid replacement, using monitors to watch hemodynamic including cerebral oximetry and using small doses of pressors or inotropes as needed to support the hemodynamics (32). The amount of ANH removed is calculated once the target hematocrit on CPB is determined and the following formula is used:

Target hematocrit on CPB =
Starting HCT [circulating blood volume (CBV)] −
Volume of ANH removed)/ (CBV + Prime volume for CPB) (32).

A large meta-analysis looking at ANH in adult world showed a clear reduction in the use of allogenic blood transfusion (33). In neonates and infants ANH is much more challenging due the small size and higher risk of hemodilution. ANH in this population is more successful when done in conjunction with other strategies and in institutions where there is a robust multidisciplinary program for blood conservation.

##### Surgical Techniques

Superior surgical skills, meticulous technique, short CPB and hypothermia time and shorter duration of procedure all play a key role in minimizing perioperative bleeding. The ability of the surgeon to adequately manage bleeding in the OR cannot be understated. Electrocautery and ultrasonic devices are commonly used to help toward achieving this goal and this along with use of passive and active topical hemostatic agents are extremely valuable. Topical agents achieve hemostasis by activating the coagulation pathway either directly or indirectly and include bovine collagen, cellulose, porcine gelatin and thrombin.

#### Perfusion Strategies During CPB

##### Circuits

Surgery for congenital heart defects in pediatric patients undergoing cardiopulmonary bypass (CPB) has induced up to a 300% hemodilutional effect due to circuit prime volumes (34). Condensing surface area and prime volume are especially important in neonatal patients where the volume from the CPB circuit can be the major determinant in the patient's metabolic response to surgery (35). This hemodilution effect due to prime volume can result in multiple blood product exposures, all of which have been shown to further increase the morbidity of CPB (36, 37). Thus, attempts to condense the circuit prime volume can result in a reduction of exposure to blood products (38). The size of the circuit and the prime volume is determined by the target cardiac index needed for “full flow” and the smallest circuit and volume needed to achieve this goal need to be utilized.

##### Retrograde Autologous Prime

This consists of replacing the CPB pump circuit volume with patients' blood. Prior to initiation of CPB the arterial and venous lines are drained into the CPB circuit and the bloodless prime is drained into a reservoir. This is a well-established and beneficial strategy in adult cardiac surgery to reduce autologous blood transfusion (39, 40). The conduct of RAP in children requires a very meticulous approach with effective communication between the perfusionist, anesthesiologist and the surgeon. It needs to be a very slow process while closely watching hemodynamics and cerebral oximetry. Any perturbation in patient's hemodynamic status will need to be corrected and it is desirable to use pressors or inotropes rather than fluids to minimize hemodilution.

##### Ultrafiltration

The fundamental objectives of ultrafiltration during pediatric cardiac surgery are to increase the concentration of red blood cells and coagulation factors, remove excess fluid and inflammatory mediators during or immediately following conclusion of CPB. There are different modalities of ultrafiltration and include conventional ultrafiltration (CUF), zero balance ultrafiltration (ZBUF) and modified ultrafiltration (MUF). CUF is used in all pediatric cardiac surgery needing CPB and involves removal of excess fluid by a hemofilter and concentrated blood returned to the venous reservoir. This is usually done intermittently to avoid excessive volume depletion. Unlike CUF, ZBUF replaces the volume of ultrafiltrate removed with crystalloid without running into circuit volume contraction, thus potentially improving or increasing the amount of inflammatory mediator removal (41, 42). MUF removes blood directly from the patient, flowing through the ultrafilter then directly returns the concentrated volume to the patient. This is usually done after patient is weaned from CPB. There are numerous studies looking at benefits of MUF in pediatric surgery. Benefits of MUF include improved pulmonary compliance and gas exchange, increased hematocrit and blood pressure levels. However, there has been questionable impact on long term benefits such as duration of intubation or intensive care unit stay (43, 44).

##### Cell Salvage

Intraoperative cell salvage is a blood conservation technique to reduce allogeneic transfusion requirements related to excessive blood loss. This technique recovers blood lost in the operative field and is then anticoagulated, washed and centrifuged in a cell-saver machine and given back to the patient. The washing is said to remove debris from shed blood thus reducing the risk of cerebral thromboembolism and improving neurological outcomes. Washing also removes platelets, coagulation factors and other plasma proteins and hence its use is controversial. The ability of the newer machines to salvage even smaller volumes of blood has enabled its use in children. Cell salvage is widely used in pediatric cardiac surgery and has shown to be safe and effective in reducing postoperative allogenic blood and blood product transfusions (45, 46).

## Blood Tests and Hemostasis

Despite all the measures outlined so far, clinicians should be prepared to deal with blood loss and coagulation abnormalities that are produced by multiple factors in pediatric heart surgery. This will include judicious and timely use of blood products and hemostatic agents guided by clinical observations, laboratory and point of care testing where available.

Peri-operative and post-operative blood tests and their frequency should be selected for value in providing information crucial to the essential care of the patient, while limiting the total volume of blood sampled for this information. Using smallest sampling tubes and volumes that maintain accuracy of testing can be helpful (neonatal tubes). There is conflicting evidence whether preoperative or intraoperative testing can predict which patients are at risk for excessive bleeding due to coagulopathy post cardiac surgery. Routine coagulation testing preop is not indicated but should be performed on a case-by-case basis (47).

A complete Blood count (CBC) and cross matching for appropriate amount of blood products is accepted practice in the majority of centers. Clinical examination, preoperative history and hospital course and family history may indicate more elaborate preoperative testing. For example, further PREOPERATIVE testing may be indicated if abnormalities are observed in routine tests, or if anemia management mandates it (Ep, iron therapy). Other indications may include known or suspected abnormal liver function, preoperative anticoagulation, history of thromboembolic phenomenon and congenital coagulopathy.

During the intraoperative pre-CPB arterial blood gas, baseline Activated Clotting Time (ACT) and heparin level are obtained. A predicted heparin response is obtained via the heparin assay.

### Heparin Management

Adequate anticoagulation is essential to prevent thrombosis in the extracorporeal circuit, a devastating complication, but it also helps blood conservation by preventing unbridled activation of the coagulation cascade, thrombin generation and consumption of coagulation factors and platelets which causes more bleeding post-CPB.

Unfractionated heparin is the preferred anticoagulant during CPB with a long history of clinical use and overall safety. It has a short half-life, reliably reversed by protamine sulfate, and unaffected by renal function. It potentiates the inhibition of antithrombin III (AT III) on thrombin and factor Xa, as well as tissue factor pathway inhibitor. Heparin-induced anticoagulation during CPB is monitored by ACT, which measures the coagulation status of whole blood. Recommended dose of 400 IU per kg is administered by central venous access and ACT is used to confirm a satisfactory response based on institutional protocols (usually 3 times the baseline or >480 s).

However, the ACT is influenced by hypothermia, platelet dysfunction and hemodilution on CPB and the gold standard is laboratory assay of plasma heparin concentration by estimating anti-Xa activity. Traditional weight-based dosing derived from adults may lead to lower than adequate heparin levels in infants because of higher hemodilution, metabolic rates and blood volume to weight ratios. This may be inadequate to prevent thrombin generation, leading to more platelet dysfunction and more postoperative bleeding. Ideally the heparin dosing and protamine reversal should be individualized for optimum anticoagulation. A useful alternative for infants is an automatic protamine-titration device for whole blood heparin concentration in the OR (Hepcon HMS, Medtronic, Minneapolis, MN). This often predicts higher heparin dosing in infants than weight-based dosing, which theoretically prevents hemostatic activation on CPB and better preservation of hemostatic functions post-CPB. A protocol based on the HMS adjusted for infants reduced bleeding in a neonatal trial (48). The accuracy and limitations of the technology (c. 1990's) and its role in this era remains a subject of debate.

Neonates and small infants are observed to have lower Antithrombin III (ATIII) level producing heparin resistance and an inadequate ACT response, placing them at higher risk for thrombin generation. This may necessitate administration of Fresh Frozen Plasma (FFP) to restore AT III levels. The Network for the Advancement of Patient Blood Management, Hemostasis and Thrombosis (NATA) recommends considering FFP in pump prime in neonates. Antithrombin III concentrates are also available.

An alternative to heparin is the direct thrombin inhibitor (DTI) Bivalirudin, which does not require AT III, has a short onset and elimination half-life (25 min) and is administered by continuous infusion. Besides direct inhibition of circulating and clot bound thrombin, it also reduces thrombin induced platelet activation. There is no specific reversal of Bivalirudin, but it is short acting and clinical effect wears off within 30–60 min of ceasing infusion. Approved for percutaneous coronary interventions, it has a role in cardiac surgery with previous adverse reactions to Heparin and Heparin Induced Thrombocytopenia (HIT) as well as anticoagulation for extracorporeal support and ventricular assisted devices. Monitoring of effect is usually by activated Partial Thromboplastin Time (aPTT) or ACT, but non-linear relationship in these tests has resulted in emergence of alternative assays like dilute thrombin time (dTT), chromogenic anti-IIa assays, and the ecarin clotting time (ECT). DTIs may cause a concentration dependent elevation in the international normalized ratio (INR) (49).

### Protamine

This should be given as a slow bolus while watching for adverse reactions. Side effects include several types of protamine reactions which can vary from mild to catastrophic (severe systemic hypotension and pulmonary hypertension necessitating returning to CPB support). Using a 1:1 reversal dosing may give rise to excessive protamine dosing, which can actually impede coagulation. A heparin assay like Hepcon HMS can be used to determine the appropriate reversal dose. Managing heparin dosing and reversal with protamine using measurement of heparin level is perhaps a better approach than ACT guided and weight-based dosing. In practice we use both simultaneously at our institution.

### Visco-Elastic Testing

Post-CPB management includes timely restoration of the coagulation system guided by clinical parameters as well as coagulation tests. Viscoelastic testing (VET) has become a useful tool in monitoring and restoration of coagulation in addition to the gold standard of coagulation tests [Prothrombin time (PT)/aPTT/INR/ Fibrinogen levels].

Both Thromboelastography (TEG; Haemonetics Corporation, Braintree, MA) and rotational thromboelastometry (ROTEM; TEM Systems Inc, Research Triangle Park, NC) have been extensively studied in adult cardiac surgery, though pediatric literature is not as robust. In their latest iterations (TEG 5000, TEG 6s or ROTEM sigma) they offer a point of care (POC) solution to provide a holistic status of the coagulation process in real time following a brief lag. In many centers the parameters of TEG or ROTEM can be visualized in the operating room continuously. These tests are based on the measuring the viscoelastic properties of the developing clot and subsequent clot lysis. Each technology has established unique normal parameters and ranges that point to the contribution of platelets, fibrinogen and other factors to clot strength as well as fibrinolytic activity. Abnormalities in these can guide specific targeted product replacement. Transfusion algorithms guided by VET and other POC tests have shown to reduce bleeding and transfusions in some pediatric studies (50, 51). In a patient where there is generalized coagulopathy observed, dynamic tests like TEG or ROTEM can supplement the cross-sectional view provided by routine tests (aPTT/ PT/INR/D-Dimer and Fibrinogen). It is debatable whether VET or other coagulation tests can predict which patients will bleed excessively. In a recent singe center study, a battery of coagulation tests, including VET, failed to add substantial predictive value to clinical factors in identifying patients who needed transfusion with products for “clinical concern for bleeding (CCB)” (52).

A recent technology addition is the Quantra QPlus System (HemoSonics LCC, Charlottesville, VA) and the newest thromboelastographic monitor from Haemonetics, the TEG 6S, that use an ultrasound-based technology, called sonic estimation of elasticity via resonance (SEER) sonorheometry which interrogates the developing clot with ultrasonic waves to obtain proprietary parameters based on resonance, defining platelet, fibrinogen and factor contribution to clot strength. These may have the advantage of ease of use based on cartridge technology and a shorter lag time before results but has undergone limited evaluation. Overall VET seems to reproduce coagulation process, suffer from some false positives but have good negative predictive values and results are not mutually interchangeable. No technology seems to be vastly superior to others (53). Whichever type is chosen; institutional transfusion algorithms should identify specific ranges for the parameters monitored in children.

### Antifibrinolytics

Fibrinolysis is triggered almost simultaneously with thrombosis (by activated Factor XII and kallikrein cleaving plasminogen to plasmin). Excessive fibrinolysis can contribute significantly to ongoing blood loss despite restoration of coagulation pathway components. Antifibrinolytic agents such as Lysine analogs, Tranexamic Acid (TXA) and Epsilon Aminocaproic acid (EACA), have been shown to reduce blood loss and transfusions in multiple surgical scenarios. Dosing regimens for pediatric patients and optimal plasma levels *in vivo* remain a subject of debate with TXA, although its efficacy is fairly well-established. Inhibition of glycine mediated inhibitory pathways by TXA, and inhibition of GABA receptors is a proposed mechanism for seizures in high doses, though it is rarely reported in pediatrics. Thromboembolic complications are a potential concern. Great variability exists in the dosing regimens across institutions as the ideal plasma concentration that blocks fibrinolysis *in vivo* is not established. Based on review of existing literature NATA guidelines propose a dosing regimen as below (47):

Children <1 year of age—TXA loading dose of 30 mg/kg followed by a continuous infusion of 10 mg/kg/h
Children >1 year of age—TXA loading dose of 10 mg/kg followed by a continuous infusion of 10 mg/kg/h can be used until the end of surgery.

### Other Evaluations

Additional labs to assess oxygen delivery (Serum lactate, Acid-Base balance, Mixed venous oxygen saturation) and general metabolic and physiological homeostasis (Vital signs, urine output, Near Infrared spectroscopy-NIRS) should be monitored to support transfusion algorithms to ensure the hematocrit and cardiac output is adequate for tissue oxygenation.

### Blood Products

#### Platelets

The cell-based model of hemostasis (54) is of the dominant view currently and highlights the contribution of platelets to secondary hemostasis. The hemostatic dysfunction on CPB is both in platelets numbers (hemodilution, damage in the extracorporeal circuit) and function (inflammatory response, platelet activation and receptor damage). Destruction on CPB circuit over long CPB times and dilution with platelet poor pump prime contribute to this. Ten to twenty mls/kg dose of group matched platelets is the logical initial step in restoring hemostatic function, if there is clinical bleeding and/or the rewarming lab platelet count is low. Platelet count and VET parameters are helpful in directing platelet transfusions along with clinical assessment.

#### Cryoprecipitate

Concentrated pooled product of plasma is high in fibrinogen (Factor II) content as well as Factor VIII. After protamine administration, ongoing bleeding with a low fibrinogen level (<150 mg/dl) or VET indications of hypofibrinogenemia should indicate Cryoprecipitate transfusion of 1 unit per 5 kg (fibrinogen 250–700 mg per unit) An alternative is to give Fibrinogen concentrate if available.

#### Fibrinogen Concentrates

As a concentrated product which has undergone pasteurization, theoretically FC offer a safe lower volume alternative to Cryoprecipitate for Hypofibrinogenemia and can be considered.

#### Fresh Frozen Plasma

Rich in Vit K dependent factors, FFP can restore both volume and probably help hemostasis in factor deficiency but is due to larger volumes required is implicated in the development of post op TRALI and is a poor source of fibrinogen. It may, however, be useful as part of the pump prime in neonates (47).

#### Prothrombin Complex Concentrates

These are available in 4-factor (4F) (FII, FVII, FIX, and FX) and 3-factor (3F) (FII, FIX, FX, and very low FVII) preparations. They have several theoretical advantages over FFP in reversing coagulopathy due to deficient Vitamin K dependent factors, low volume required, less infection risk and rapid reversal and have been used anecdotally in children with excessive bleeding or in small series, but concerns remain about thrombotic risk. A recent observational study of 50 children (0–6 years) early use of fibrinogen, platelets and PCCs based on clinical bleeding score and VET reduced bleeding and improved hemostasis with no complications (55). The European Society of Cardiothoracic Anesthesia (EACTA) consensus statement on (adult) use of 4 factor PCCs highlights lack of safety data and recommends reduced dosing (25 IU per KG). If they are to be used for refractory bleeding, outside the labeled indications, ensuring normal fibrinogen levels (required for their effect) and adequate antithrombin III is essential (56). However, as the evidence on pediatric dosing, risk of thrombosis and adequate effect monitoring is lacking, NATA recommendations are against using them outside of a clinical trial.

##### Factor VIII Inhibitor Bypassing Activity

FIEBA is another PCC containing FII, FIX, FX, and activated VIIa. It is approved for hemophilia A and B patients with high inhibitor levels and has been used anecdotally in pediatric cardiac surgery for excessive bleeding. For example in a single center review, recombinant Factor VIIa and FEIBA use resulted in reduction in post cardiac surgery bleeding in children and adults, but was associated with thrombotic phenomena (57). Similar to other PCCs, the concerns about dosing, monitoring of effect and thrombotic concerns preclude widespread recommendation (47).

#### Factor VIIa

Recombinant activated factor VIIa (rFVIIa; NovoSeven, Novo Nordisk, Copenhagen, Denmark) is indicated for the treatment of bleeding in Hemophilia with inhibitors and aids in thrombin generation.

There are multiple case reports and small series of successful use of these hemostatic agents in pediatric heart surgeries. This is balanced by ongoing concerns about thromboembolic complications, appropriate dosing and clear indications (58).

It is expected that PCCs, FEIBA and Factor VIIa will continue to be used in an off-label fashion in the face of excessive ongoing bleeding when usual measures are exhausted. These patients should be carefully monitored for thrombotic phenomena.

#### Umbilical Cord Blood

An intriguing option is to use autologous umbilical cord blood (collected at birth in prenatally diagnosed CHD) for autotransfusion in neonatal heart surgery. Although anecdotal cases exist of the plausibility, issues of collection, storage, timing of surgery (amongst others) need clarification as evidence is gathered to establish its future role (59).

## Summary

While often necessary due to the nature and complexity of cardiac surgery, there is increasing evidence that transfusion of blood components is not benign and may lead to adverse outcomes in both adults and children following heart surgery. Guidelines are available for patient blood management in adult cardiac surgery but their widespread adoption in practice is uncertain (60). There is a paucity of high-level evidence in pediatrics to establish similar guidelines, but these efforts are ongoing (61). NATA (www.nataonline.com) has come up with evidence-based recommendations after an expert review of the available evidence (47). The strength of evidence supporting their recommendations is modest (B/C), but such efforts may help create institutional Patient Blood Management (PBM) programs or transfusion algorithms that adapt to clinical and local circumstances. Several studies suggest that the implementation of transfusion algorithms may optimize utilization and improve outcomes in pediatric cardiac surgery.

## Author Contributions

Both Authors share a lot of interest in the topic and has contributed throughout.

## Conflict of Interest

The authors declare that the research was conducted in the absence of any commercial or financial relationships that could be construed as a potential conflict of interest.

## Publisher's Note

All claims expressed in this article are solely those of the authors and do not necessarily represent those of their affiliated organizations, or those of the publisher, the editors and the reviewers. Any product that may be evaluated in this article, or claim that may be made by its manufacturer, is not guaranteed or endorsed by the publisher.
